# A plasma cytokine and angiogenic factor (CAF) analysis for selection of bevacizumab therapy in patients with metastatic colorectal cancer

**DOI:** 10.1038/srep17717

**Published:** 2015-12-01

**Authors:** Long Bai, Feng Wang, Dong-sheng Zhang, Cong Li, Ying Jin, De-shen Wang, Dong-liang Chen, Miao-zhen Qiu, Hui-yan Luo, Zhi-qiang Wang, Yu-hong Li, Feng-hua Wang, Rui-hua Xu

**Affiliations:** 1 Department of Medical Oncology, Sun Yat-sen University Cancer Center, Guangzhou, Guangdong 510060, P. R. China; 2 State Key Laboratory of Oncology in South China; Collaborative Innovation Center for Cancer Medicine, Guangzhou, Guangdong 510060, P. R. China; 3 Department of Medical Oncology, Zhejiang Cancer Hospital, Hangzhou 310022, P. R. China

## Abstract

This study intends to identify biomarkers that could refine the selection of patients with metastatic colorectal cancer (mCRC) for bevacizumab treatment. Pretreatment 36 plasma cytokines and angiogenic factors (CAFs) were first measured by protein microarray analysis in patients who received first-line bevacizumab-containing therapies (discovery cohort, n = 64), and further evaluated by enzyme-linked immunosorbent assay in patients treated on regimens with or without bevacizumab (validation cohort, n = 186). Factor levels were correlated with clinical outcomes, predictive values were assessed using a treatment by marker interaction term in the Cox model. Patients with lower pretreatment levels of hepatocyte growth factor (HGF) or VEGF-A^121^ gain much more benefit from bevacizumab treatment as measured by progression-free survival (PFS) and overall survival (OS), while angiopoietin-like 4 (ANGPTL4) levels negatively correlated with PFS and response rate following bevacizumab (all adjusted interaction *P* < 0.05). A baseline CAF signature combining these three markers has greater predictive ability than individual markers. Signature-negative patients showed impaired survival following bevacizumab treatment (PFS, 7.3 vs 7.0 months; hazard ratio [HR] 1.03; OS, 29.9 vs 21.1 months, HR 1.33) compared with signature-positive patients (PFS, 6.5 vs 11.9 months, HR 0.52; OS, 28.0 vs 55.3 months, HR 0.67). These promising results warrant further prospective studies.

Angiogenesis was a critical component in carcinogenesis and tumor growth, and therapies targeting tumor angiogenesis were major steps in the treatment of metastatic colorectal cancer (mCRC)^1^. Bevacizumab, the vascular endothelial growth factor-A (VEGF-A) monoclonal antibody, was the first proven target agent in the treatment of metastatic colorectal cancer (mCRC) and currently was a standard of care for mCRC patients in combination with first-line chemotherapy^2^,^3^,^4^. Despite the overall success of bevacizumab, clinical efficacy was variable and some individuals seemed to be resistant against it, resulting in rather modest gains in overall survival (OS) under bevacizumab-containing therapies^5^,^6^,^7^. Thus, the identification of predictive biomarkers will be urgently required in achieving the full therapeutic potential of bevacizumab.

Whereas mutations in the K-RAS oncogene well predicted resistance to epidermal growth factor receptor (EGFR) monoclonal antibodies in mCRC treatment^8^,^9^,^10^, equivalent reliable predictors of bevacizumab were currently lacking^11^,^12^. Possible baseline predictors of bevacizumab included circulating short VEGF-A isoforms and expression of VEGF receptors (VEGF receptor-1 [VEGFR1] or neuropilin 1 [NRP1]), either in plasma or in tumor tissues^13^,^14^. Beyond that, most biomarker studies were limited by the small sample size or the nonrandomized nature. The single-arm study design was unable to distinguish between markers associated with general prognosis for the cytotoxic chemotherapy component of the regimen and markers that predict for benefit afforded by bevacizumab. Furthermore, none of them has been consistently replicated across different studies and cancer types^15^,^16^,^17^,^18^. To overcome some of the deficiencies of earlier investigations, we simultaneously assessed a series of cytokines and angiogenic factors (CAFs) with a commercially available protein microarray which incorporated various markers known to be associated with the effect of bevacizumab in previous studies, first in pretreatment plasma of patients who received first-line bevacizumab-containing therapies from a single-center registry study, and next in patients treated on a regimen with or without bevacizumab. We also explored a “CAF index” that combined individual markers to better serve as a signature for predicting benefit from bevacizumab.

## Methods

### Patients and study design

This study has designed two cohorts of patients. The discovery cohort population was developed from data source in a single-center registry study, which was initiated in 2012 January for evaluating the efficacy and safety profile of bevacizumab combined with first-line chemotherapy in Chinese mCRC patients at Sun Yet-sen University Cancer Center. The recorded patients were assigned to treatment with backbone chemotherapies of FOLFIRI^19^ (45.6%), FOLFOX^20^ (34.9%), or XELOX^21^ (19.5%), in combination with bevacizumab 5 mg/kg every 2 weeks (5-FU-based regimens) or 7.5 mg/kg every 3 weeks (capecitabine-based regimens). The standardized data collection form which was adopted after each cycle of treatment, has recorded individual demographic characteristics, co-morbidities, laboratory data, dates and doses of bevacizumab therapy, treatment pattern, clinical effectiveness and adverse events, from the first cycle until completion of bevacizumab.

From the data source, a total of 178 histological proven locally advanced or metastatic CRC patients were screened for study eligibility. Enrolled patients should have received at least 3 cycles of first-line bevacizumab administration, have no prior chemotherapy for metastatic disease and with at least 6 months elapsed from completion of any adjuvant chemotherapy or radiotherapy. Patients were excluded if they had any surgery within one month from the collection of blood sample, brain metastases, or without adequate follow-up. Written informed consent was obtained from each patient regarding the use of plasma for this translational research.

A prespecified limit for hazard ratio of 0.54 was chosen to detect a difference in progression-free survival (PFS) of 6 months (7/13 months) or more, 64 events were required for a one-sided significance level of 10% and a statistical power of 80% (East Software; Cytel, Cambridge, MA). Hence 64 mCRC patients in Sun-Yet Sen University Cancer Center were recruited by systematic sampling from the registry. For the validation cohort, the sample size was chosen to maximize statistical power, thus this validation cohort utilized all patients recorded in the registry study, except for the patients chosen as the discovery cohort (hereinafter referred to as the bevacizumab group); with a random selection of a cross-section of mCRC patients (approximately 1:1) who received a combination treatment of chemotherapy but without bevacizumab at the Sun Yet-sen University Cancer Center by systematic sampling (hereinafter referred to as the control group).

### Biomarker assessments and methodology

The pretreatment peripheral venous blood sample were first subjected to analysis with the RayBio^®^ Human Angiogenesis Antibody Array 2 (RayBiotech Inc; Norcross, Georgia, USA) according to the manufacturer’s instructions^22^. To reduce variation between slices, all plasma samples were screened simultaneously and each unique target factor was assessed in quadruplicate. These factors were given alphabetically in Supplementary Table S1. The protein microarray utilized the sandwich-ELISA design principle. Briefly, samples were incubated with the arrays and then with a cocktail of biotinylated detection antibodies, followed by a streptavidin-conjugated fluor. Signals were visualized using a fluorescence laser scanner and saved digitally. The resulting value was the globally normalized densitometric value (GNDV) of the loci, the averaged GNDV value of the quadruplicates of each factor was referred to hereafter as the mean densitometric value (MDV), and was the value used for final interpretation of data in our analyses (details in supplementary experimental procedure and data analysis).

Promising candidate markers in the CAF profiling were further measured from the validation cohort by commercially available ELISA method per the manufacturers’ directions^23^. Antibodies against ANGPTL4 and HGF were purchased from R&D (Minneapolis, MN, USA), and anti-VEGF-A^121^ antibody was from Cusabio (Wuhan, Hubei, China). Samples were allocated onto the arrays using a randomized block design and analyzed in duplicate with no more than one prior freeze-thaw cycle and the mean value was used for final analysis. Out-of-range value was substituted with the median value of that analyte, and measurements below the detection level were conventionally assigned a value equal to half of the detection threshold. On retrospective review of computed tomography imaging, blood samples at the point of best response or disease progression (henceforth denoted as “at response” and “at progression”) were also measured by ELISA test. The study has been carried out in compliance with the Helsinki declaration, the protocol has been approved by the Institutional Review Board (IRB) and Human Ethics Committee of Sun Yet-sen University Cancer Center. Informed consent was obtained from all subjects involved in this study.

### Statistical analysis and CAF signature development

The categorical data in this report were summarized by frequencies and percentages, the continuous covariates by median, range, and numbers of observations. Parameters others than survival were compared using the Mann–Whitney test and the Chi-square-test. Correlations between biomarker levels and the following clinical parameters were assessed in current study: OS, defined as time between initiation of first-line treatment and death from any cause; PFS, defined as time between initiation of first-line treatment and disease progression or death; overall response rate (ORR), including partial response (PR) and complete response (CR) per Response Evaluation Criteria in Solid Tumors criteria version 1.1 (RECIST 1.1)

Upon microarray profiling, the CAF analytes were initially explored as continuous variables and subsequently categorized as binary variables (using the median MDV as cut-off). Univariate analysis was performed among CAFs according to their baseline MDVs, by Cox regression for PFS and OS, and by logistic regression for ORR. Because of the exploratory nature of this step, no adjustment for multiple testing was made.

Candidate analytes in the CAF profiling were subsequently subjected to ELISA test to validate the predictive worth. Hazard ratios (HRs) for PFS and OS, and odds ratios (ORs) for ORR were calculated according to baseline analytes levels (dichotomized at the optimal cut-off) and treatment arm. The interaction between treatment effects and analyte concentration was assessed by interaction Wald tests from Cox proportional hazards model (for PFS and OS) and logistic regression models (for ORR) to determine the predictive value. The *maximum statistic approach* was used to search for appropriate cut-off point. This procedure considers all possible values of the continuous covariate as potential cut-points. The optimal cut-point is the value of the continuous covariate that gives the maximum different degrees of benefit from bevacizumab (i.e. that with the smallest interaction *P*-value)^24^,^25^. Additional multivariate regression models that incorporated treatment arm, biomarker, the interaction between these two, and clinical prognostic variables were also run. We next established a CAF “index” containing these candidate markers with the goal of identifying groups of patients who experienced different degrees of benefit from bevacizumab. Similarly, interaction Cox hazards model was used to test the predictive value of the CAF signature.

The primary end-point was the PFS, with secondary end-point being OS and ORR for all analyses. Kaplan–Meier plots and log-rank tests were applied to illustrate and compare patients’ survival. Pairwise correlations among analytes were evaluated by Spearman’s rank test. To assess the dynamic changes of analytes levels between different time points, parameters were compared by paired-sample t test or Wilcoxon matched-samples rank sum test depending on the normality of the on-treatment changes. All statistical analyses were performed using SPSS 17.0 (SPSS, Chicago, IL, USA) and STATA 12.0 (Stata, College Station, TX, USA).

## Result

### Patients

**Figure 1:**
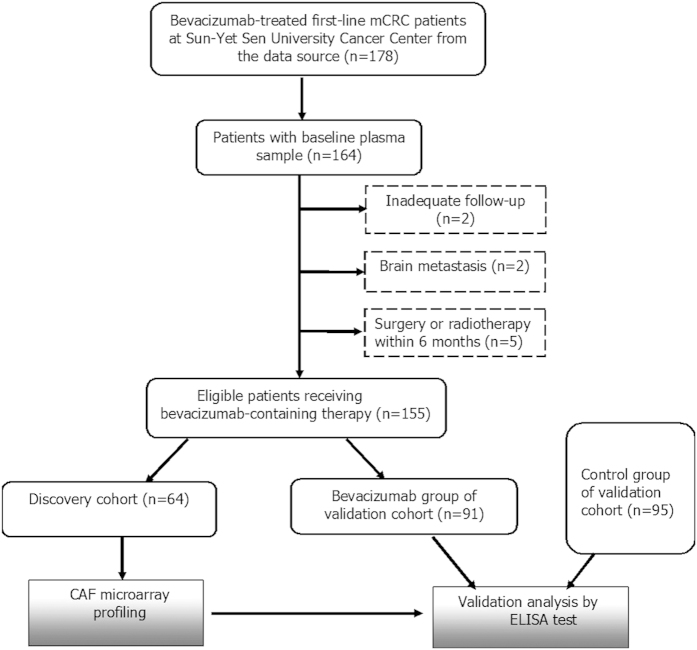
Study Patient Disposition; CAF, cytokine and angiogenic factor.

Among the 178 mCRC patients considered for this study from the registry data source, 155 patients were deemed eligible on the basis of inclusion and exclusion criteria from December 2011 to April 2014. Specifically, except for the 64 patients selected as the discovery cohort, the other 91 patients were chosen as the bevacizumab group of validation cohort. In addition, a cross-section of 95 patients treated at Sun Yat-sen University Cancer Center who had a similar chemotherapy but without bevacizumab were chosen as the control group of validation cohort (Fig. 1). Furthermore, 12 and 11 plasma samples acquired from the bevacizumab group were available at the time of tumor response and radiographic progression, respectively.

As of February 2015, after a median follow-up of 21.5 months (range, 1.5–40.6 months), median PFS, median OS, and ORR were 8.8 months (95% CI: 7.3–10.1 months), 24.6 months (21.7–27.4) and 45.2% in the discovery cohort population. Meanwhile, median PFS and OS were 10.5 months (9.1–11.8) and 26.6 months (20.6–32.5) in the bevacizumab group versus 6.9 months (5.7–8.1) and 24.5 months (20.5–28.4) in the control group, while ORR was 41.4% in bevacizumab-group and 31.0% in the control group.

The demographic, clinical, and pathologic characteristics of patients in the bevacizumab group were similar to those in the control group, as well as to patients incorporated in the discovery cohort (Table 1).

### Baseline CAF profile

**Figure 2:**
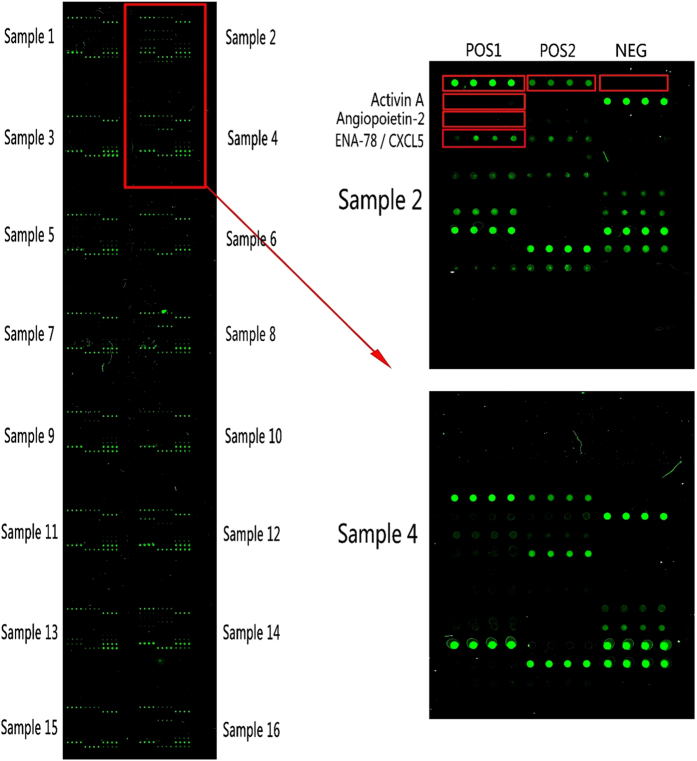
A typical individual slice of protein microarray, using a panel of 36 cytokines from RayBio® Human Angiogesis Antibody Array 2, the images were captured using a GenePix® 4000A scanner, a total of 8 slices (2 slices for each patient) were processed to generate data for 64 patients. POS1, positive control 1; POS2, positive control 2; NEG, negative control; ENA-78/CXCL5, neutrophil-activating peptide 78/chemokine (C-X-C motif) ligand 5.

Figure 2 represents one of the microarray slices. The MDVs of bFGF, HB-EGF and TPO were below detection threshold in more than 20% patients and was not further analyzed for clinical parameter relevance.

In univariate analysis, PFS was longer in patients with lower MDVs of hepatocyte growth factor (HGF; HR 1.84, 95% CI 1.05–3.24, *P* = 0.034), VEGF-A^121^ (HR 1.85, 95% CI 1.11–3.08, *P* = 0.019), angiopoietin-like 4 (ANGPTL4; HR 1.64, 95% CI 0.97–2.79, *P* = 0.06) and groucho (GRO; HR 1.64; 95% CI 0.97–2.75, *P* = 0.077) than patients with high MDVs. While Activin-A (HR 0.47, 95% CI 0.27–0.82, *P* = 0.019) was inversely correlated with PFS (Table 2 and Fig. S1). OS was remarkably prolonged in patients with low MVD levels of ANGPTL4, HGF and Angioprotein-2 (Ang-2). Lower MDVs of ANGPTL4, HGF, VEGF^121^ and Ang-2 were correlated with better ORR. Therefore, ANPGLT4, HGF and VEGF-A121 correlated with all assessed outcome parameters when analyzed as both continuous (for each standard deviation change) and discrete variables (median as the cut-off), and were further evaluated by ELISA.

### Biomarker levels by ELISA test and correlations with clinical characteristics

The lower limits of detection for ANGPTL4, HGF and VEGF-A^121^ were 0.25 ng/mL, 0.2 ng/mL and 0.1 ng/mL, respectively. Pre-therapeutic plasma concentrations of ANGPTL4 ranged from 0.28 to 140.5 ng/mL (median: 2.4 ng/mL); HGF, 0.26 to 2.51 ng/mL (median: 0.8 ng/mL) and VEGF-A^121^, 0.12 to 18.5 ng/mL (median: 0.6 ng/mL). Assays were highly reproducible with intra-assay and inter-assay coefficients of variation (CV) for ANGPTL4 being 2.1% and 6.4%; HGF, 1.3% and 4.3%; VEGF-A^121^, 8.2% and 10.5%.

Weak association (correlation coefficients between 0.2 and 0.4) and very weak association (correlation coefficients between 0 and 0.19) were noted for 2 pairs of analytes: ANGPTL4-VEGF-A^121^ (0.33, *P* < 0.001), and HGF-VEGF-A^121^ (0.19, *P* = 0.011), respectively. There were no noteworthy correlations between baseline levels of ANGPTL4-HGF (0.01, *P* = 0.996). Baseline levels of these analytes were unbiased by demographic and clinical characteristics except that VEGF^121^ levels were slightly higher in patients who had primary tumor resected than those who had not (median, 0.87 *v* 0.59 ng/L; *P* = 0.045; Mann-Whitney *U* test) (Supplementary Table S2).

### Predictive value of baseline factors on the benefits with bevacizumab by ELISA test

Table 3 shows the clinical outcomes by treatment separately for groups defined by low and high baseline levels. For disease progression, the HRs were <1 in most subcategories, indicating the superiority of bevacizumab-containing treatment over chemotherapy alone. Therefore, we searched for markers that identified groups of patients who experienced different degrees of benefit from bevacizumab, using cutoff values determined by *maximum statistic approach*.

**Figure 3:**
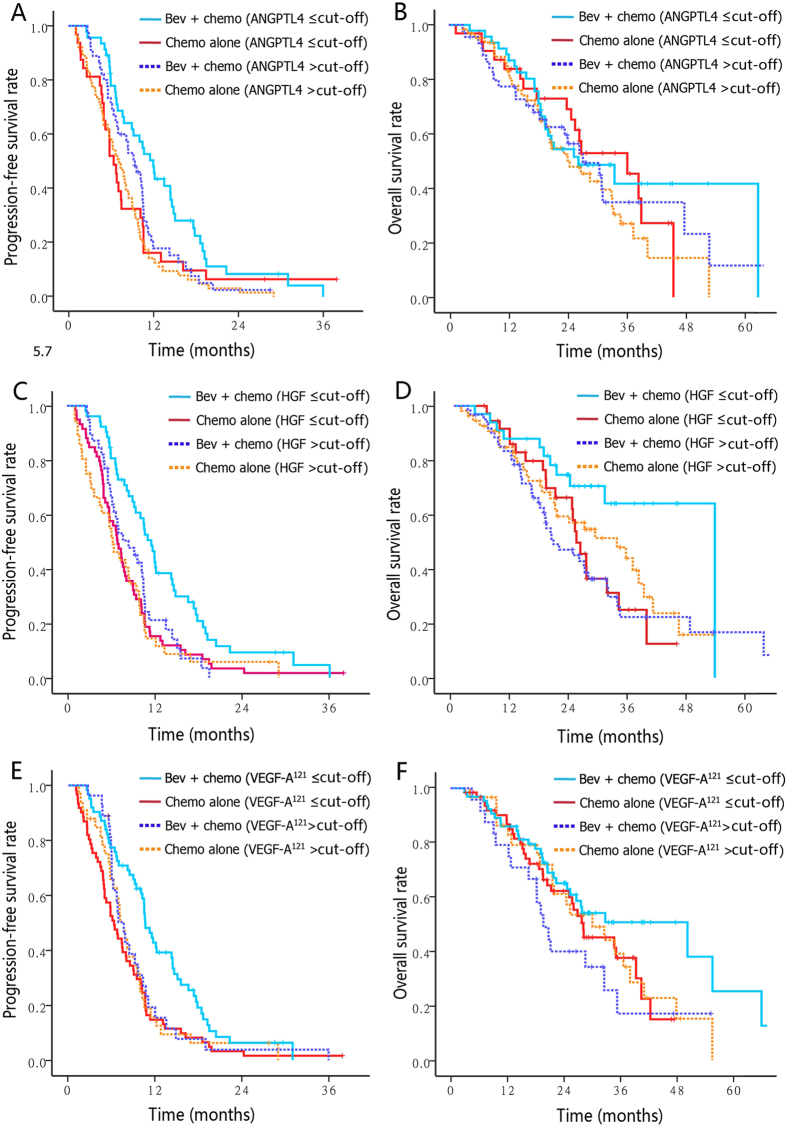
The predictive value of candidate markers for progression-free survival (A, C, E) and overall survival (B, D, F) in ELISA test were presented by Kaplan–Meier curves stratified according to baseline marker levels (using corresponding optimal binary split) and treatment arms. BEV, bevacizumab; chemo, chemotherapy.

**Figure 4:**
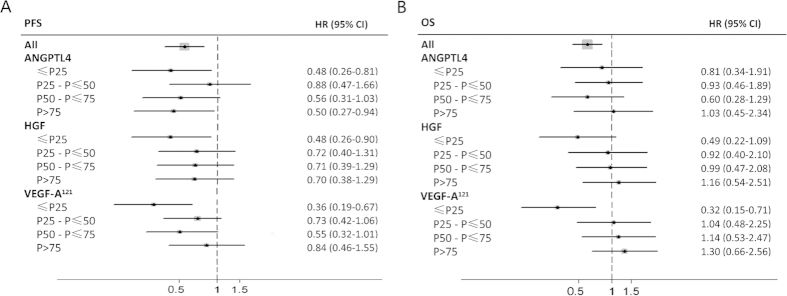
Forest plots of hazard ratios (bevacizumab plus chemotherapy versus chemotherapy alone) for (A) PFS and (B) OS by each biomarker (categorised into quarters). PFS, progression-free survival; OS, overall survival; HR, hazard ratio 95% CI, 95% confidence interval; ANGPTL4, angiopoietin-like 4; HGF, hepatocyte growth factor; VEGF-A^121^, isoform vascular endothelial growth factor-A^121^.

As a result, patients with lower baseline plasma levels of ANGPTL4 (i.e. lower than the median 1.97 ng/ml), HGF (0.88 ng/ml) or VEGF^121^ (0.59 ng/ml) were more sensitive to bevacizumab treatment as measured by PFS (for ANGPTL4, HR 0.58; 95% CI 0.36–0.94; for HGF, HR 0.52, 95% CI 0.35–0.76; for VEGF121, HR 0.52; 95% CI 0.36–0.76) than those with higher analytes levels (for ANGPTL4, HR 0.73; 95% CI 0.49–1.08, *unadjusted P* = *0.048* from interaction Cox Wald tests; for HGF, HR 0.79, 95% CI 0.49–1.27, unadjusted interaction *P* = *0.020*; for VEGF^121^, HR 0.90; 95% CI 0.53–1.53, unadjusted interaction *P* = *0.023*). Similarly, patients with lower baseline HGF or VEGF-A^121^ levels experienced remarkably larger benefit from bevacizumab in terms of OS than patients with higher analytes levels (for HGF, HR 0.42 versus 1.19, lower versus higher levels, unadjusted interaction *P* = 0.010; for HGF, HR 0.42 versus 1.25, lower versus higher levels, unadjusted interaction *P* = 0.034) (Table 3 and Fig. 3). With the addition of bevacizumab, response rates were only increased in patients with lower ANGPTL4 levels (48.8% vs 10.7%) rather than those with higher ANGPTL4 levels (34.1% vs 41.1%; unadjusted interaction *P* = *0*.003). Likewise, patients who had lower HGF or VEGF^121^ levels also showed a trend toward improved ORR versus patients with higher levels, though the interaction test did not show enough power (Table 3). The *P*-values for treatment-marker interaction remained significant in multivariate models after adjusted for known clinical prognostic variables (gender, age, performance status, primary tumor site, tumor grade, prior adjuvant chemotherapy, number of metastasis site, and curative-intent metastasis resection) (Table 3). To identify trends that may not have been apparent at the binary split, analytes were further categorized and analyzed by quartile. The forest plots provided a clear trend indicating that the outcomes became poorer as the concentrations of these markers increased, patients with baseline VEGF^121^ or HGF concentrations in the lowest quartile obtained the most survival benefit from bevacizumab (Fig. 4).

From baseline to the point of disease progression, plasma HGF level was significantly increased (mean concentration, 0.85 ng/mL to 1.15 ng/mL, *P* = 0.047). Meanwhile, ANGPTL4 increased and VEGF-A^121^ decreased in 9 of 11 patients, respectively. But the change did not reach statistical significance (3.0 to 8.2 ng/mL, *P* = 0.15 for ANGPTL4; 0.76 to 0.54 ng/ml, *P* = 0.07 for VEGF-A^121^). Nevertheless, none of these factors showed significant changes from baseline to best tumor response (ANGPTL4, 2.9 to 3.9 ng/mL, p = 0.16; HGF, 0.79 to 0.75 ng/mL, *P* = 0.60; VEGF-A^121^, 0.60 to 0.39 ng/mL, *P* = 0.26).

**Figure 5:**
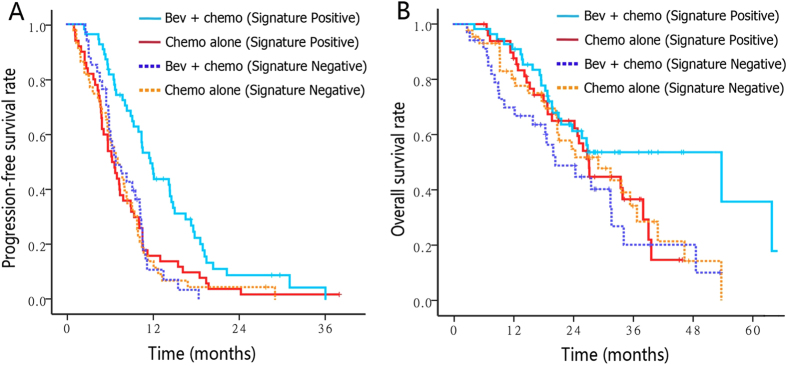
PFS (A) and OS (B) Kaplan–Meier plots of patients stratified by treatment arm based on three-marker signature index (positive versus negative). The signature negative indicates CAF Index = 0 or 1; the signature positive indicates CAF Index = 2 or 3. A score of +1 was assigned for candidate marker (ANGPTL4, HGF or VEGF^121^) concentrations below the corresponding cut-off or 0 for those above the cut-off. Then the score of each marker was added for each patient. Bev, bevacizumab; Chemo, chemotherapy.

To create a “CAF index” from these candidate markers (ANGPTL4, HGF or VEGF^121^), a score of +1 was assigned for markers concentrations below the corresponding cut-off or 0 for those above the cut-off. Then the index for each patient was calculated by adding the score for each marker so that the final CAF index ranged from 0 to 3. We selected a CAF index value of 1 as the cut-off because the resulting groups were the most balanced (Half patients with CAF Index = 0 or 1 [signature negative] and half with CAF Index = 2 or 3 [signature positive]). Signature negative individuals showed no improvement in PFS following bevacizumab treatment (7.3 vs 7.0 months; HR, 1.03) compared to signature positive individuals (6.5 vs 11.9 months; HR, 0.52). With the addition of bevacizumab, median OS was only prolonged in patients with positive signature (28.0 vs 55.3 months; HR 0.67), while signature negative patients had an impaired OS following bevacizumab (29.9 vs 21.1; HR 1.33) (Fig. 5). The interaction between the CAF signature and the effect of bevacizumab was highly significant (interaction P = 0.001 for PFS; interaction P = 0.011 for OS).

## Discussion

This study aims to search for circulating biomarkers that could identify patients likely to benefit from bevacizumab. Finally, patients with either lower baseline ANGPTL4, HGF or VEGF-A^121^ levels experienced the most benefit from addition of bevacizumab to chemotherapy for PFS (42%, 48% and 47% reduction in HRs, respectively). The three-marker CAF index could further refine the selection of patients expected to benefit or lack of benefit from bevacizumab (interaction P = 0.001 for PFS; interaction *P* = 0.011 for OS).

Factors known to be associated with the effect of bevacizumab in prior studies were either not incorporated in our study (VEGF-A, vascular cell adhesion molecule 1 [VCAM-1], intercellular cell adhesion molecule-1 [ICAM-1], E-selection, matrix metalloproteinase-2 [MMP-2])^16^,^26^,^27^, or failed to predict benefit of bevacizumab (Angiopoietin-2, Insulin-like growth factor 1 [IGF-1], soluble VEGFR1, soluble VEGFR2, heparin-binding epidermal growth factor [HB-EGF], basic fibroblast growth factor [bFGF], IL-8, platelet-derived growth factor beta polypeptide b [PDGF-BB] and placental growth factor [PlGF])^27^,^28^,^29^, although ANG-2 potentially associated with survival (Table 2).

VEGF-A, the target of bevacizumab, was the intuitive candidate predictor for bevacizumab therapy^30^. Beyond its well-established prognostic value, the use of VEGF-A for antiangiogenic treatment outcome prediction remains uncertain^31^,^32^,^33^. Baseline levels of VEGF-A have fail to predict the effectiveness of bevacizumab in most investigations involving multiple cancer types, and the increased levels of VEGF-A was deemed to be indicator of poor prognosis of mCRC regardless of whether bevacizumab was part of the therapy^26^,^34^,^35^,^36^,^37^.

Recent data suggested that a modified ELISA with a preference to detect short VEGF-A isoforms might be more promising in discovering bioactive VEGF-A. These isoforms freely diffused over long distances, thereby provided more specific readout of tumor-secreted VEGF-A^38^,^39^. Several trials have revealed that patients with metastatic breast cancer, gastric cancer or pancreatic cancer who expressed high pretreatment levels of VEGF-A^121^ would exhibit improved PFS and/or OS after bevacizumab treatment, but the results have been inconsistent and failed to replicate in similar research for CRC, NSCLC and RCC^16^,^39^,^40^. In current study, patients with low baseline VEGF-A^121^ levels could derive more benefit from bevacizumab treatment as measured by PFS and OS relative to patients with high VEGF-A^121^ levels (Table 3; Figs 3 and 4), largely owing to the more complete blockage of the ligand at lower VEGF-A^121^ concentrations. These different results across studies suggested that the roles of VEGF-A^121^ may be context and cancer type-dependent. The phase III MERiDiAN trial which evaluated the impact of bevacizumab in metastatic breast cancer patients stratified for plasma VEGF-A^121^ will confirm the definitive predictive value of VEGF-A^121^.

HGF and its receptor, the MET tyrosine kinase, was frequently deregulated in cancer cells and promoted its proliferation, invasion and migration^41^,^42^, the expression of the HGF/c-Met in serum or tumor has been reported to play important roles in the progression and prognosis of solid tumors^43^,^44^,^45^. The predictive effect of baseline HGF level under VEGF-targeting therapies has not been implicated in mCRC before. With respect to other VEGFR tyrosine kinase inhibitors (TKIs), low baseline HGF level has been reported to correlate with better clinical outcomes in pazopanib-treated patients with renal cell carcinoma (RCC) or soft-tissue sarcoma^28^,^46^. Moreover, HGF has been reported to be a potent angiogenic factor via activated HGF/c-Met pathway and may augment the VEGF expression under hypoxia conditions^47^,^48^. On the contrary, VEGF on endothelial cells can lead to increased expression of HGF and subsequent activation of MET on tumor cells, thereby resulting in a paracrine feedback loop between tumor and vasculature^49^,^50^. Additionally, recent research has demonstrated that VEGF could negatively regulate HGF/c-MET axis through a MET/VEGFR2 heterocomplex, therefore suppress tumor invasion^51^. Hence, we hypothesized that in malignant phenotypes with enhanced HGF/c-Met signaling, the therapeutic VEGF blockade could restore and increased MET activity, thus overcame the inhibitory effects of bevacizumab. Above data suggested it might be able to select mCRC patients upfront who may likely to have primary bevacizumab resistance by assessing circulating HGF and VEGF-A^121^ levels. A subset of patients might benefit from treatment modalities that concurrently block VEGF and HGF/Met signaling^51^,^52^.

ANGPTL4 was identified in systemic circulation and share amino acid sequence similarity with the angiopoietin family (ANGs)^53^. Recent findings have revealed ANGPTL4 as a critical factor in tumor growth and progression in cancers^54^,^55^,^56^,^57^ and a potential therapeutic target^58^,^59^,^60^. Several new studies shown that ANGPTL4 might exert a VEGF-independent proangiogenic effect, meanwhile, impair VEGF-induced neovascularization^59^,^61^,^62^,^63^. Thus, we speculated that higher ANGPTL4 expression could represent an alternative tumor angiogenesis route which was resistant to VEGF inhibition, hence lead to a poor therapeutic effect of bevacizumab.

Peak levels at the emergence of radiographic disease progression was seen with HGF and VEGF-A^121^, indicated that upregulation of VEGF-A^121^ and HGF may act as a mechanism of adaptive resistance against bevacizumab, and may be used as early markers for disease progression after anti-VEGF therapy. The pairwise correlation between pretreatment VEGF-A^121^ and HGF levels, and correlation between ANGPTL4 and VEGF^121^ levels were rather weak. Further investigation may provide insights into the coregulation and counter regulation of these factors and their underlying biology.

Our study first reported the impact of pre-therapeutic ANGPTL4 and HGF concentrations on the benefit of bevacizumab, and the upregulation of VEGF-A^121^ and HGF may be used as early markers for disease progression after anti-VEGF therapy. Furthermore, combining multiple angiogenic biomarkers to generate predictive signatures is a highly promising strategy. We need to acknowledge the inherent bias within this retrospective study, and the exploratory results from relative small sample size should be interpreted with caution. Ideally, plasma markers offer the ability to carry out continuous assessments over time, are easy to standardize and reproduce and may be more relevant to the metastatic tumor being treated than to the tissue-based measurements in archival primary tumors. Nevertheless, the type of plasma analyzed, the response of non-neoplastic, host tissue to the tumor, and other preanalytic factors, such as the number of freeze–thaw cycles could affect the circulating cytokine levels^13^,^18^. Moreover, angiogenesis is a complex and highly adaptive biologic process, the microarray system used in current study only assess part of the CAFs, multiple other factors could also play essential roles during angiogenesis^13^. A better understanding of the underlying cellular and molecular mechanisms of ANGPTL4, HGF, and VEGF-A^121^ will reveal the potential therapeutic value of these factors. Above all, these promising results warrants further prospective study to confirm their values in order to customize combined chemotherapeutic and anti-angiogenic treatment.

## Additional Information

**How to cite this article**: Bai, L. *et al*. A plasma cytokine and angiogenic factor (CAF) analysis for selection of bevacizumab therapy in patients with metastatic colorectal cancer. *Sci. Rep.* **5**, 17717; doi: 10.1038/srep17717 (2015).

## Supplementary Material

Supplementary Information

## Figures and Tables

**Table 1:** Baseline demographics and clinical characteristics across different treatment groups.

Clinical characteristics *N (*%)	Discovery Cohort	Validation Cohort		
		Bevacizumab group	Control group	*P*-value^a^
No. of patients	64	91	95	**—**
Age at diagnosis	52 (26–77)	53 (21–76)	55 (26–83)	0.93
Sex				0.45
male	35 (56.5)	59 (60.2)	56 (63.6)	
female	27 (43.5)	39 (39.8)	32 (36.4)	
Location of primary tumor				0.54
colon	42 (67.7)	62 (68.1)	60 (63.2)	
rectum	20 (32.3)	29 (31.9)	35 (36.8)	
ECOG performance status				0.94
0–1	84 (95.5.)	92 (97.9)	84 (95.5)	
≥2	4 (4.5)	2 (2.1)	4 (3.5)	
Pathology				0.73
moderately differentiated	40 (65.6)	55 (69.6)	60 (73.2)	
poorly differentiated	21 (34.4)	24 (30.4)	22 (26.8)	
Matestasis				0.30
single	31 (50.0)	46 (50.5)	56 (58.9)	
multiple	31 (50.0)	45 (49.5)	39 (41.1)	
Curative-intent matestasis resection	6 (9.7)	17 (18.7)	19 (20.0)	0.86
Backbone chemotherapy				0.28
oxaliplatin-based	36 (56.2)	63 (70.0)	57 (61.3)	
irinotecan-based	28 (43.8)	27 (30.0)	36 (38.7)	
Prior adjuvant chemotherapy	17 (27.4)	30 (31.6)	23 (25.8)	0.42
Recurrent disease^b^	17 (27.4)	23 (25.3)	31 (32.6)	0.33
First-line duration of CT (median, mos)	5 (3.0–18.0)	4.5 (1.5- 25.0)	3.8 (2.5–24.0)	0.18
Total duration of CT (median, mos)	12 (3.0–22.0)	9.5 (2.0–25.0)	11.5 (2.5–26.0)	0.60
Second-line treatment	23 (39.0)	17 (23.3)	14 (15.9)	0.27
Anti-EGFR treatment following first PD	3 (4.8)	22 (24.7)	31 (32.6)	0.26
Maintenance treatment^c^	20 (32.3)	10 (13.3)	14 (15.6)	0.83

^a^Compared patient characteristics between the bevacizumab group and the control group.

^b^Patients who had metastatic disease more than 6 months elapsed from adjuvant chemotherapy.

^c^Monotherapy of capecitabine or bevacizumab, or combined with both. Abbreviations: ECOG PS, eastern cooperative oncology group performance status; EGFR, epidermal growth factor receptor; CT, chemotherapy; mos, months. Statistical significance was set at 0.05 based on a two-sided test. *P*-values listed in bold were notable for possible association with clinical outcomes.

**Table 2:** CAFs with significant or borderline prognostic values in protein microarray profiling.

Biomarkers	Progression-Free Survival^b^			Overall Survival^b^			Overall Response Rate^b^		
	Median, mos	HR (95% CI)	*P*	Median, mos	HR (95% CI)	*P*	ORR (%)	OR (95% CI)	*P*
Activin-A									
Low vs High^a^	6.8 vs 10.6	0.47 (0.27–0.82)	**0.007**	21.1 v 31.7	0.54 (0.26–1.12)	**0.098**	41.9 vs 48.4	1.30 (0.48–3.54)	0.610
Each SD change	—	1.09 (0.77–1.53)	0.637	—	0.74 (0.32–1.69)	0.473	—	0.40 (0.07–2.19)	0.293
ANGPTL4									
Low vs High^a^	10.5 vs 6.2	1.64 (0.97–2.79)	**0.061**	32.0 vs 24.6	2.45 (1.07–5.61)	**0.034**	67.2 vs 26.1	0.16 (0.05–0.55)	**0.004**
Each SD change	—	1.24 (0.95–1.63)	**0.096**	—	1.73 (1.22–2.49)	**0.002**	—	0.13 (0.03–0.66)	**0.014**
Ang-2									
Low vs High^a^	10.6 vs 7.1	1.24 (0.73–2.10)	0.430	27.5 vs 20.7	1.88 (0.92–3.82)	0.189	56.4 vs 34.8	0.30 (0.11–0.86)	**0.025**
Each SD change	—	1.12 (0.84–1.49)	0.439	—	1.40 (0.95–2.07)	**0.089**	—	0.38 (0.13–1.17)	**0.092**
GRO									
Low vs High^a^	10.1 vs 7.1	1.64 (0.97- 2.75)	**0.077**	27.5 vs 18.3	1.70 (0.85–3.40)	0.134	58.1 vs 32.3	0.54 (0.32–1.17)	**0.073**
Each SD change	—	1.02 (0.82–1.28)	0.835	—	0.85 (0.55–1.34)	0.490	—	1.13 (0.68–1.86)	0.636
HGF									
Low vs High^a^	12.1 vs 5.7	1.84 (1.05- 3.24)	**0.034**	32.7 vs 19.0	2.62 (1.09–5.68)	**0.017**	61.5 vs 28.6	0.25 (0.08–0.78)	**0.017**
Each SD change	—	1.18 (0.96–1.44)	**0.091**	—	1.34(1.01–1.80)	**0.047**	—	0.28 (0.10–0.82)	**0.020**
VEGF-A^121^									
Low vs High^a^	10.6 vs 6.1	1.85 (1.11–3.08)	**0.019**	25.2 vs 20.7	1.46 (0.73–2.91)	0.278	64.3 vs 35.7	0.34 (0.12–0.97)	**0.044**
Each SD change	—	1.50 (1.16–1.94)	**0.002**	—	1.17 (0.85–1.59)	0.337	—	0.49 (0.18–1.30)	0.152

^a^Patients were dichotomized according to the median mean densitometric value (MDV) of each molecule. High indicates above the median MDV; low indicates less than or equal to the MDV.

^b^Cox regression analysis was performed to test different clinical outcomes between the lower and upper median MDV or between each standard deviation (SD) increase in MDV. Abbreviations: ORR, overall response rate; ANGPTL4, angiopoietin-like 4; HGF, hepatocyte growth factor; Ang-2, angiopoietin-2; VEGF-A^121^, isoform vascular endothelial growth factor-A ^121^. OR, odds ratio; 95% CI, 95% confidence interval. Statistical significance was set at 0.10 based on a two-sided test. Other footnotes as in Table 1.

**Table 3:** Correlations between baseline biomarker levels and clinical outcomes by ELISA test.

Biomarker	Progression-Free Survival^a^				Overall Survival^a^				Overall Response Rate^a^			
	Median (mos)	HR (95% CI)	*P*	*P*^b^	Median (mos)	HR (95% CI)	*P*	*P*^b^	Median (%)	OR (95% CI)	*P*	*P*^b^
All patients	6.9 v 10.5	0.60 (0.44–0.81)	0.001	—	24.5 v 26.6	0.80 (0.56–1.15)	0.230	—	31.0 v 41.4	1.58 (0.84–2.96)	0.20	—
ANGPTL4												
Low	6.5 v 12.2	0.58 (0.36–0.94)	0.023	**0.054** (**0.041**)	37.9 v 27.5	0.96 (0.51–1.80)	0.900	0.596 (0.980)	10.7 v 48.8	8.00 (2.09–30.3)	0.002	**0.003**
High	7.3 v 9.5	0.73 (0.49–1.08)	0.115		25.7 v 28.5	0.84 (0.50–1.41)	0.520		41.1 v 34.1	0.74 (0.33–1.69)	0.476	
HGF												
Low	7.0 v 11.7	0.52 (0.35–0.76)	0.001	**0.020** (**0.039**)	26.0 v 55.3	0.42 (0.20–0.89)	0.020	**0.010** (**0.026**)	28.8 v 49.0	2.37 (1.04–5.38)	0.040	0.134
High	6.3 v 8.5	0.79 (0.49–1.27)	0.323		34.5 v 21.1	1.19 (0.74–1.90)	0.473		34.4 v 31.6	0.88 (0.32–2.40)	0.804	
VEGF-A^121^												
Low	6.4 v 10.8	0.53 (0.36–0.76)	0.001	**0.023** (**0.028**)	28.0 v 50.0	0.42 (0.21–0.84)	0.011	**0.034** (**0.032**)	28.1 v 39.3	1.66 (0.77–3.60)	0.136	0.141
High	7.8 v 7.8	0.90 (0.53–1.53)	0.70		29.9 v 19.6	1.25 (0.77–2.02)	0.370		37.0 v 46.2	1.46 (0.49–4.37)	0.583	

^a^Patients were stratified according to baseline marker levels (using corresponding optimal cutoff), clinical outcomes of control group versus bevacizumab group were compared using Cox proportional hazards model (for PFS and OS), and logistic regression models (for ORR). High indicates above the corresponding cutoff and low indicates less than or equal to the corresponding cutoff. The cutoff values for ANGPTL4, HGF and VEGF121 were 1.97 ng/ml, 0.88 ng/ml and 0.59 ng/ml, respectively.

^b^*P*-value for treatment-marker interaction assessed by interaction Wald Cox proportional hazards model (for PFS and OS), and interaction logistic regression models (for ORR). In parentheses, the interaction *P*-value was adjusted for known clinical prognostic variables (gender, age, performance status, primary tumor site, tumor grade, prior adjuvant chemotherapy, number of metastasis site, and curative-intent metastasis resection). Abbreviations: ELISA, enzyme-linked immunosorbent assay. Statistical significance was set at 0.05 based on a two-sided test. Other footnotes as in Table 1.
