# Supplementary material for: A plasma cytokine and angiogenic factor (CAF) analysis for selection of bevacizumab therapy in patients with metastatic colorectal cancer
Source: Sci Rep. 2015 Dec 1;5:17717. doi: 10.1038/srep17717 (PMC4664961; doi:10.1038/srep17717)
Supplement: Supplementary Information [file srep17717-s1.pdf]

A plasma cytokine and angiogenic factor (CAF) analysis for selection of bevacizumab therapy in patients with metastatic colorectal cancer

Long Bai[1,2*], Feng Wang[1,2*], Dong-sheng Zhang[1,2], Cong Li[3], Ying Jin[1,2], De-shen Wang[1,2], Dong-liang Chen[1,2], Miao-zhen Qiu[1,2], Hui-yan Luo[1,2], Zhi-qiang Wang[1,2], Yu-hong Li[1,2],Feng-hua Wang [1,2 §], Rui-hua Xu[1,2 §]

Authors' Affiliations: [1] Department of Medical Oncology, Sun Yat-sen University Cancer Center; [2] State Key Laboratory of Oncology in South China; Collaborative Innovation Center for Cancer Medicine, Guangzhou, Guangdong 510060, P. R. China; [3] Department of Medical Oncology, Zhejiang Cancer Hospital, Hangzhou 310022, P. R. China.

[§]Corresponding Author: Rui-hua Xu, Department of Medical Oncology, Sun Yat-sen University Cancer Center, 651 Dongfeng Road East, Guangzhou, Guangdong 510060, P. R. China. Tel: +86-20-87343468; Email: xurh@sysucc.org.cn. Wang-feng Hua, Department of Medical Oncology, Sun Yat-sen University Cancer Center, 651 Dongfeng Road East, Guangzhou, Guangdong 510060, P. R. China. Tel: +86-20-87343795; Email: wangfh@sysucc.org.cn.

*These authors contributed equally to the manuscript.

Table S1. 36 baseline CAFs analyzed in protein microarray profiling

Activin A	IFN gamma	PIGF
AgRP/ART	IGF-1	RANTES / CCL5
Angiogenin (ANG)	IL-1 alpha	TGF beta 1
Angiopoietin-1 (ANG-1)	IL-2	TIMP-1
Angiopoietin-2 (ANG-2)	IL-6	TIMP-2
ANGPTL4	IL-8 / CXCL8	TNF alpha
bFGF / FGF-2	IL-17	TNF beta
ENA-78 / CXCL5	IP-10 / CXCL10	TPO
ESM-1 / endocan	Leptin	VEGF-A^{121}
GRO (CXCL 1,2&3)	LIF	sVEGFR1
HB-EGF	MCP-1 / CCL2	sVEGFR2
HGF	PDGF-BB	vWF

Abbreviations: CAFs, cytokine and angiogenic factors; AgRP/ART, agouti-related protein/agouti-related transcript; ANGPTL4, angiopoietin-like 4; bFGF/FGF-2, basic fibroblast growth factor/fibroblast growth factor 2; ENA-78/CXCL5, neutrophil-activating peptide 78/chemokine (C-X-C motif) ligand 5; ESM-1, endothelial cell specific molecule-1; GRO (CXCL 1,2&3), groucho (chemokine [C-X-C motif] ligand 1,2&3); HB-EGF, heparin-binding EGF-like growth factor; HGF, hepatocyte growth factor; IFN gamma, interferon gamma; IGF-1, insulin-like growth factor 1; IL-1 alpha, interleukin-1 alpha; IP-10- interferon-gamma-inducible protein 10; LIF, leukaemia Inhibitor Factor; MCP-1/CCL2, monocyte chemotactic protein 1/chemokine (C-C motif) ligand 2; PDGF-BB, platelet-derived growth factor beta polypeptide b; PIGF, placental growth factor; TGF beta 1, transforming growth factor beta 1; TIMP-1, metallopeptidase inhibitor 1; TNF alpha, tumor necrosis factor alpha; TPO, thrombopoietin; VEGF-A^{121}, short isoform vascular endothelial growth factor-A^{121}; sVEGFR1, soluble vascular endothelial growth factor receptor-1; vWF, von Willebrand factor.

Table S2. Baseline biomarker levels and associations with clinical characteristics by ELISA test

Clinical characteristics *N (%)*	ANGPTL4			VEGF-A[121]			HGF		
	Low [a]	High [a]	*P* [b]	Low [a]	High [a]	*P* [b]	Low [a]	High [a]	*P* [b]
Treatment groups			0.055			0.531			0.551
bevacizumab group	46 (48.9)	48 (51.1)		62 (65.3)	33 (34.7)		52 (57.1)	39 (42.9)	
control group	32 (34.8)	60 (65.2)		61 (69.3)	27 (30.7)		59 (62.1)	36 (37.9)	
Median age at diagnosis	55 (28-78)	53 (21-83)	0.294	53 (21-77)	56 (25-83)	0.465	54 (28-83)	55 (21-78)	0.625
Sex			0.170			0.521			0.759
male	53 (46.1)	62 (53.9)		78 (69.0)	35 (31.0)		70 (60.9)	45 (39.1)	
female	25 (35.2)	46 (64.8)		45 (64.3)	25 (35.7)		36 (54.5)	30 (45.5)	
Primary tumor			0.213			0.740			0.433
colon	47 (38.5)	75 (61.5)		80 (66.1)	41 (33.9)		70 (57.4)	52 (42.6)	
rectum	31 (50.0)	31 (50.0)		43 (69.4)	19 (30.6)		41 (64.1)	23 (35.9)	
ECOG performance status			0.734			0.361			0.302
0-1	73 (41.5)	103 (58.5)		116 (66.7)	58 (33.3)		107 (60.8)	69 (39.2)	
⩾2	3 (50.0)	3 (50.0)		3 (60.0)	2 (40.0)		2 (33.3)	4 (66.7)	
Pathology			0.600			0.085			0.291
moderately differentiated	47 (40.9)	68 (59.1)		73 (64.0)	41 (36.0)		71 (61.7)	44 (38.3)	
poorly differentiated	21 (45.7)	25 (54.3)		35 (79.5)	9 (20.5)		24 (52.2)	22 (47.8)	
Metastasis			0.456			0.430			0.098
single	40 (39.2)	62 (60.8)		70 (70.0)	30 (30.0)		55 (53.9)	47 (46.1)	
multiple	38 (45.2)	46 (54.8)		53 (63.9)	30 (36.1)		56 (66.7)	28 (33.3)	

Peritoneal metastasis	24 (47.1)	27 (52.9)	0.408	34 (68.0)	16 (32.0)	0.74	32 (62.7)	19 (37.3)	0.620
Metastasis resection	18 (42.9)	24 (57.1)	0.680	29 (69.0)	13 (31.0)	0.955	25 (59.5)	17 (40.5)	0.966
Resection of primary tumor	61 (43.3)	80 (56.7)	0.604	99 (71.2)	40 (28.2)	**0.045**	84 (59.6)	57 (40.4)	1.000
Backbone chemotherapy			0.800			0.859			0.233
oxaliplatin-based	50 (41.7)	70 (58.3)		79 (64.2)	39 (35.8)		75 (62.5)	45 (37.5)	
irinotecan-based	27 (42.9)	36 (57.1)		44 (71.0)	18 (29.0)		34 (54.0)	29 (46.0)	
Prior adjuvant chemotherapy	27 (50.9)	26 (49.1)	0.138	37 (71.2)	15 (28.8)	0.600	34 (64.2)	19 (35.8)	0.508
Recurrent disease	24 (44.4)	30 (55.6)	0.744	36 (67.9)	17 (32.1)	1.000	29 (53.7)	25 (46.3)	0.325
Maintenance treatment [c]	10 (41.7)	14 (58.3)	1.000	16 (66.7)	8 (33.3)	0.944	12 (50.0)	12 (50.0)	0.368
Second-line treatment	12 (40.0)	18 (60.0)	0.611	22 (73.3)	8 (26.7)	0.551	20 (66.7)	10 (33.3)	0.472
Anti-EGFR treatment after PD	25 (47.2)	28 (52.8)	0.325	37 (71.2)	15 (28.8)	0.600	30 (56.6)	23 (43.4)	0.620
First-line duration of CT			0.882			0.161			0.655
Short [d]	38 (40.9)	55 (59.1)		58 (62.4)	35 (37.6)		55 (57.9)	40 (42.1)	
Long [d]	40 (43.0)	53 (57.0)		65 (72.2)	25 (27.8)		56 (61.5)	35 (38.5)	
First-line duration of BEV			0.532			0.495			1.000
Short [e]	21 (46.7)	24 (53.3)		29 (65.9)	15 (34.1)		26 (57.8)	19 (42.2)	
Long [e]	25 (54.3)	21 (45.7)		33 (73.3)	12 (26.7)		56 (60.9)	20 (43.5)	

[a] High indicates above the corresponding cutoff and low indicates less than or equal to the corresponding cutoff. The cutoff values for ANGPTL4, HGF and VEGF121 were 1.97 ng/ml, 0.88 ng/ml and 0.59 ng/ml, respectively. [b] Examine correlations between patient characteristics and biomarker levels. [c] Monotherapy of capecitabine or bevacizumab, or combined with both. [d] High indicates above the median first-line duration of CT; low indicates less than or equal to the median. The median first-line chemotherapy duration was 4.5 months in the bevacizumab group and 3.8 months in the control group. [e] High indicates above the median first-line duration of BEV; low indicates less than or equal to the median. The median first-line BEV duration was 4.0 months in the bevacizumab group.
Abbreviations: ELISA, enzyme-linked immunosorbent assay; CT, chemotherapy; BEV, bevacizumab; Statistical significance was set at 0.05 based on a two-sided test. *P*-values listed in bold were notable for possible association with clinical outcomes.

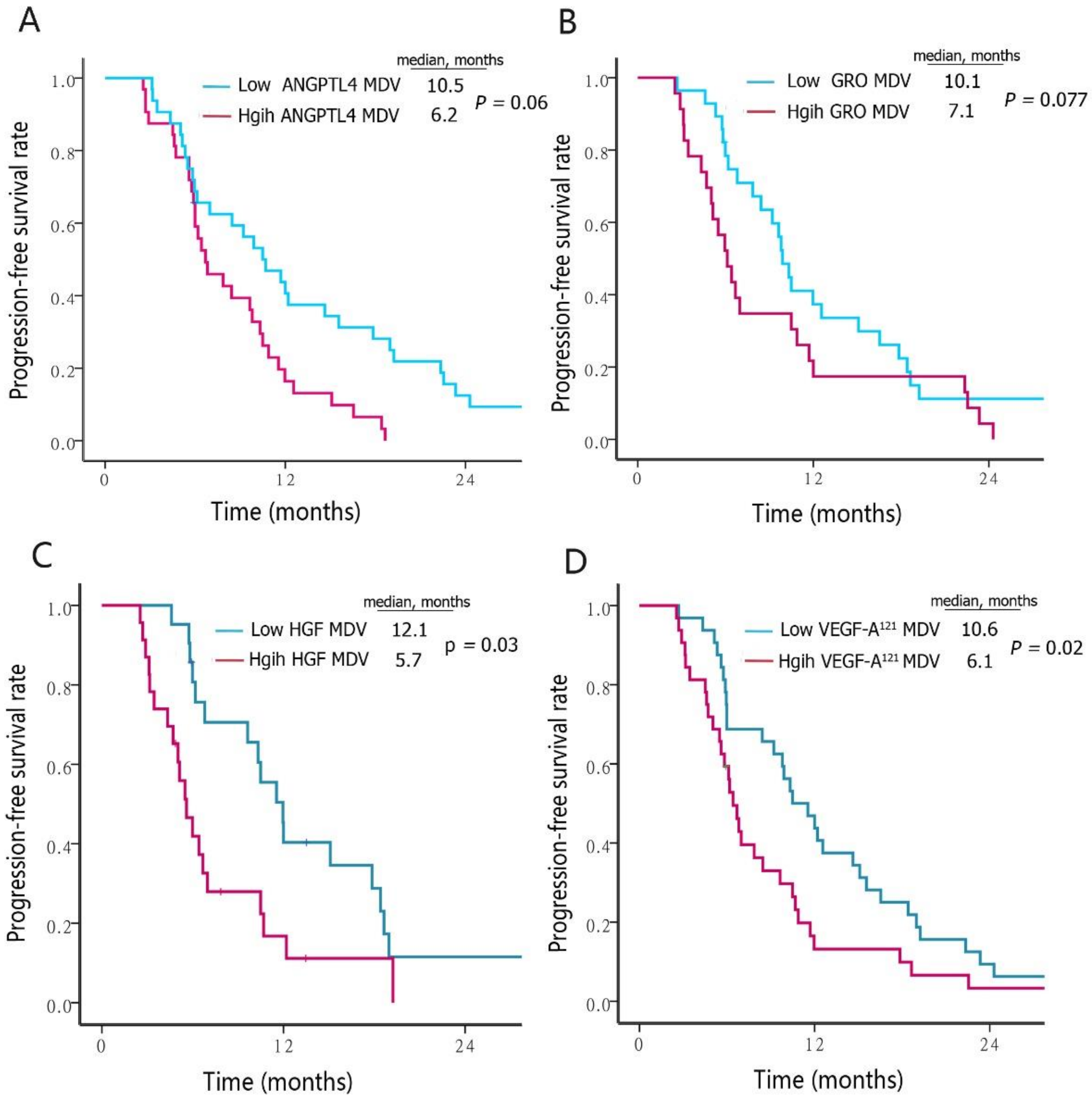

Figure S1. CAFs with significant or borderline prognostic values in protein microarray profiling were categorised as binary variables (using each median MDV as cutoff) for illustration of PFS. MDV, mean densitometric value; CAF, cytokine and angiogenic factor; ANGPTL4, angiopoietin-like 4; GRO (CXCL 1,2&3), groucho (chemokine [C-X-C motif] ligand 1,2&3); HGF, hepatocyte growth factor; VEGF-A^{121}, isoform vascular endothelial growth factor-A ^{121}.

Supplementary experimental procedure and data analysis

Plasma sample collection

4 milliliters pretreatment peripheral venous blood from each patient was drawn into a sodium citrated Vacutainer tube (BD Biosciences, San Jose, CA; catalog #369714). After mixing, the tubes were centrifuged at 2500 g for 15 minutes for separation of plasma and mononuclear cell layers. The upper layer of plasma was transferred to a fresh tube and centrifuged one more time at 2500g for 15 minutes. Then the plasma samples were aliquoted and immediately stored at − 80 °C until use. Before analysis, samples were thawed overnight at 4°C and centrifuged at 1,500 × g to remove debris.

Protein microarray profiling

The standard-sized histology slides were pre-coated with antibodies against target proteins that anchored onto the slides for sandwich-ELISA detection. Next, slides were individually placed in chambers of 16-well tissue culture plates and blocked with the kit's blocking buffer. After the blocking step, plasma diluted 1:10 was added in blocking buffer. After 2 hours of incubation, slides were repeatedly washed and then incubated with biotin-conjugated antibodies for 2 hours. Following a further set of washes, horse radish peroxidase conjugated streptavidin was added and incubated for 1 hour followed by a final set of washes to washed off nonspecific proteins

The slices were then placed in the manufacturer's chemiluminescence detection buffer and incubated for 2 minutes. Slices were subsequently exposed to with adhesive film (included in kit). The film was then captured using a GenePix® 4000A high resolution scanner (Molecular Devices; Sunnyvale, Orleans, USA) and saved digitally. The densitometric value of each locus on the array was finally measured

using a ImageJ software (National Institutes of Health; Bethesda, Maryland, USA). Subsequently, normalization was performed to account for variations between one slice and another to permit valid cross slice comparisons. Briefly, the mean value of the manufacturer's positive control replicates on each slice, called the loading control densitometric value (LCDV), was determined. The LCDV value for each membrane was subtracted from all experimental marker densitometric values on that slice. The resulting value is referred to as the loading control normalized densitometric value (LCNDV) of the marker. Hence, a second normalization was done for each plasma sample by taking the mean densitometric value of all 36 experimental markers for that sample and subtracting it from LCNDV. The resulting value is the globally normalized densitometric value (GNDV) of the loci. The GNDV values of the quadruplicates for each marker were averaged. This value was referred to as the mean densitometric value (MDV) of each cytokine, and was the value used for interpretation of data.
